# Construction of Bi_2_WO_6_/g-C_3_N_4_ Z-Scheme Heterojunction and Its Enhanced Photocatalytic Degradation of Tetracycline with Persulfate under Solar Light

**DOI:** 10.3390/molecules29051169

**Published:** 2024-03-06

**Authors:** Yukun Li, Haiyang Zhang, Dan Zhang, Sen Yao, Shuying Dong, Qishi Chen, Fengjuan Fan, Hongyuan Jia, Mingjia Dong

**Affiliations:** 1 School of Energy and Environmental Engineering, Zhongyuan University of Technology, Zhengzhou 450007, China2022108376@zut.edu.cn (F.F.);; 2 Science and Technology Innovation Coordination Service Center of Laiwu District, Jinan 271100, China; 3 MOE Key Laboratory of Yellow River and Huai River Water Environmental and Pollution Control, Henan Key Laboratory for Environmental Pollution Control, School of Environment, Henan Normal University, Xinxiang 453007, China

**Keywords:** photocatalyst, solar light, Bi_2_WO_6_/g-C_3_N_4_, persulfate, tetracycline

## Abstract

Z-scheme heterojunction Bi_2_WO_6_/g-C_3_N_4_ was obtained by a novel hydrothermal process; its photocatalysis–persulfate (PDS) activation for tetracycline (TC) removal was explored under solar light (SL). The structure and photoelectrochemistry behavior of fabricated samples were well characterized by FT-IR, XRD, XPS, SEM-EDS, UV-vis DRS, Mott-Schottky, PL, photocurrent response, EIS and BET. The critical experimental factors in TC decomposition were investigated, including the Bi_2_WO_6_ doping ratio, catalyst dosage, TC concentration, PDS dose, pH, co-existing ion and humic acid (HA). The optimum test conditions were as follows: 0.4 g/L Bi_2_WO_6_/g-C_3_N_4_ (BC-3), 20 mg/L TC, 20 mg/L PDS and pH = 6.49, and the maximum removal efficiency of TC was 98.0% in 60 min. The decomposition rate in BC-3/SL/PDS system (0.0446 min^−1^) was 3.05 times higher than that of the g-C_3_N_4_/SL/PDS system (0.0146 min^−1^), which might be caused by the high-efficiency electron transfer inside the Z-scheme Bi_2_WO_6_/g-C_3_N_4_ heterojunction. Furthermore, the photogenerated hole (h^+^), superoxide (O_2_•^−^), sulfate radical (SO_4_•^−^) and singlet oxygen (^1^O_2_) were confirmed as the key oxidation factors in the BC-3/SL/PDS system for TC degradation by a free radical quenching experiment. Particularly, BC-3 possessed a wide application potential in actual antibiotic wastewater treatment for its superior catalytic performance that emerged in the experiment of co-existing components.

## 1. Introduction

Global freshwater resources are being further depleted due to climate change, increasing demand and poor management, and many regions have been suffering from severe water shortages [1]. It was estimated that one billion urban people would face a water resources shortage worldwide by 2050 [2]. The shortage of water resources is being paid close attention all over the world, and water pollution is more seriously aggravating the problem. In particular, antibiotics have become one of the primary water pollution sources for its overuse in agricultural, biological, and medical fields, to name a few. Previous studies reported that antibiotics were detected not only in surface water and wastewater, but also in groundwater and drinking water [3]. The presence of antibiotics in the water environment posed potential harm to animals and human health, which has been drawing the broad attention of environmentalists. Further, microbial resistance genes could be activated by antibiotics [4], which made the biochemical waste water treatment process less effective and more expensive. In addition, the administration of antibiotic-containing wastewater was difficult with conventional wastewater treatment techniques such as filtration, precipitation, and disinfection, on account of its chemical stability [5]. Therefore, it is particularly important to explore effective techniques to treat antibiotic wastewater.

Advanced persulfate oxidation technology was widely used to treat antibiotic wastewater because of its strong oxidizing property, fast reaction, high stability and excellent adaptability. However, the sulfate radical (SO_4_•^−^) generated slowly from peroxymonosulfate (PMS) or persulfate (PDS), which was unfavorable to its applications in environmental purification [6]. In recent years, various methods had been developed to enhance the generation of SO_4_•^−^ including thermal [7], UV, metal ions, and the inorganic nonmetallic nanomaterial [8]. Given its ability to utilize green solar energy, the photocatalytic activation technique was enormously studied in the effluent treatment [9]. It was reported that the graphite phase carbon nitride (g-C_3_N_4_) possessed the ability to activate persulfate for TC decomposition [10]. Besides, g-C_3_N_4_ was widely used in photocatalytic technology for the characteristics of an outstanding chemical stability, suitable energy band structure, and excellent light absorption [11]. In consequence, the g-C_3_N_4_-combined light catalyst-activated persulfate was an interesting topic. However, the electron-hole (e^−^-h^+^) pairs that were generated by light, recombined readily, which hampered its practical applications [12]. A number of methods, such as metal loading, microstructure control and heterostructure construction, were used to strengthen the activity of g-C_3_N_4_ recently [13]. Constructing a heterojunction was proved to be a feasible technique among the above methods. Bi_2_WO_6_, a ternary metal oxide, has aroused much concern for its visible-light-driven performance and preeminent oxidizing ability. The Bi_2_WO_6_/g-C_3_N_4_ heterojunction constructed by Bi_2_WO_6_ and g-C_3_N_4_ could be divided into two categories (Z-scheme and type-Ⅱ), and the Z-scheme heterojunction had a better redox ability in its photocatalytic reaction than that of type-Ⅱ [14]. It was reported that constructing the Bi_2_WO_6_/g-C_3_N_4_ heterojunction was beneficial to the removal of 2,4-dichlorophenol [15]. Bi_2_WO_6_/g-C_3_N_4_ obtained by Zhao [16] via a hydrothermal reaction for 24 h exhibited good photocatalytic decomposition activity for ciprofloxacin, tetracycline, and other antibiotics under sunlight. The g-C_3_N_4_/Bi_2_WO_6_ heterojunction, with ethylene glycol used as a solvent, was synthesized through a hydrothermal reaction for 24 h [17]. However, the use of harmful solvents constricted the further development of the g-C_3_N_4_/Bi_2_WO_6_ heterojunction. It is necessary to find a novel synthesis method of energy conservation and environmental protection for the Bi_2_WO_6_/g-C_3_N_4_ heterojunction. Moreover, Bi_2_WO_6_/g-C_3_N_4_ used as the activator of PDS might be more effective for antibiotic removal under solar light (SL), which has been rarely studied.

In the present study, the Bi_2_WO_6_/g-C_3_N_4_ heterojunction was prepared by a single-step hydrothermal reaction for 3 h. The physicochemical and photoelectrochemical performances of Bi_2_WO_6_/g-C_3_N_4_ were well analyzed. The effects of different systems, Bi_2_WO_6_ doping ratio, catalyst dosage, TC concentration, PDS dose, initial pH, co-existing anions, and HA on the decomposition of TC in BC-3/SL/PDS systems were studied in detail. Furthermore, free radical quenching experiments were used to explore the dominant active species.

## 2. Results and Discussion

### 2.1. Structure and Morphology Analyses

#### 2.1.1. FT-IR Analysis

**Figure 1:**
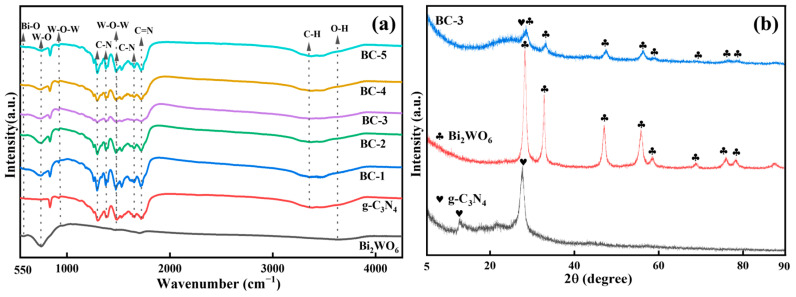
(**a**) FT-IR spectra of Bi_2_WO_6_, g-C_3_N_4_, BC-1, BC-2, BC-3, BC-4, and BC-5 composites and (**b**) XRD patterns of g-C_3_N_4_, Bi_2_WO_6_, and BC-3. Clubs and Hearts signs are used to distinguish the characteristic peaks of Bi_2_WO_6_ and g-C_3_N_4_.

The molecular information of the prepared samples was studied with FT-IR spectroscopy (Figure 1a). The peaks of Bi_2_WO_6_ at 580, 752, and 915 cm^−1^ were from the vibrations of Bi-O, W-O, and W-O-W bonds, respectively [18]. The broad peak at 3346 and 3647 cm^−1^ belonged to the C-H and O-H stretching vibration of samples [19]. In addition, the characteristic stretching patterns between 1299 and 1720 cm^−1^ were related to the C-N and C=N. The peaks at 1292, 1382, 1480, and 1640 cm^−1^ were identified as the C-N vibrations [20]. The feature peak at 1710 cm^−1^ was specified as the C=N of g-C_3_N_4_ [21]. The triazine structure peak (960 cm^−1^) was part of W-O-W. The results of the FT-IR spectroscopy demonstrated the successful synthesis of the Bi_2_WO_6_/g-C_3_N_4_ [20].

#### 2.1.2. XRD Analysis

The crystalline form of the photocatalyst was detected by XRD (Figure 1b). Pure Bi_2_WO_6_ exhibited feature peaks at 2θ = 28.3°, 32.8°, 47.1°, 55.8°, 58.5°, 68.7°, 75.9°, and 78.3°, which was consistent with the russellite phase of Bi_2_WO_6_ [22]. The peaks of pure g-C_3_N_4_ at 2θ of 12.8° and 27.7° agreed with the results reported in the literature [23]. The peaks of Bi_2_WO_6_ were precisely observed in BC-3. However, the characteristic peak of g-C_3_N_4_ was almost invisible behind the shelter of the strong peak of Bi_2_WO_6_ at 28.3° [24]. It could be concluded that BC-3 was successfully obtained.

#### 2.1.3. XPS Analysis

**Figure 2:**
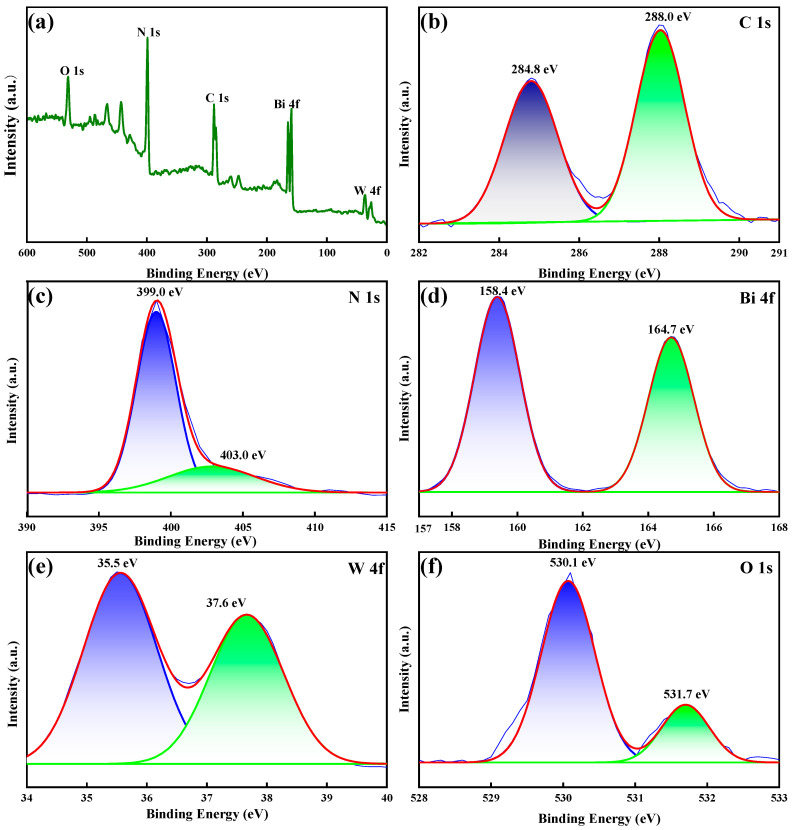
XPS spectra of BC-3: (**a**) full survey spectrum, (**b**) C 1s, (**c**) N 1s, (**d**) Bi 4f, (**e**) W 4f, and (**f**) O 1s.

The element information and structure state of the BC-3 composite were analyzed by XPS. As shown in Figure 2a, XPS spectra displayed that BC-3 was composed of carbon (41.3%), nitrogen (22.7%), oxygen (8.3%), tungsten (7.8%), and bismuth (19.9%). As revealed in Figure 2b, the C 1s spectrum could be deconvolved into two characteristic peaks at 284.8 (C-C) and 288.0 eV (C-C=N) [25]. Figure 2c illustrated the N 1s spectrum, and the peak at 399.0 eV was attributed to N-(C)_3_, while the peak (403.0 eV) was ascribed to C-N-H [23]. As shown in Figure 2d, the trait peaks at 158.4 and 164.7 eV indicated the existence of Bi^3+^ [26]. The peaks of W 4f at 35.5 and 37.6 eV belonged to W^6+^ (Figure 2e) [27]. The spectrum of O 1s (Figure 2f) showed a two-state peak (530.1 and 531.7 eV), which agreed with W-O and Bi-O, respectively [28]. The findings revealed an effective electron transfer in BC-3 and the formation of heterojunction structures.

#### 2.1.4. FE-SEM and EDS Analysis

**Figure 3:**
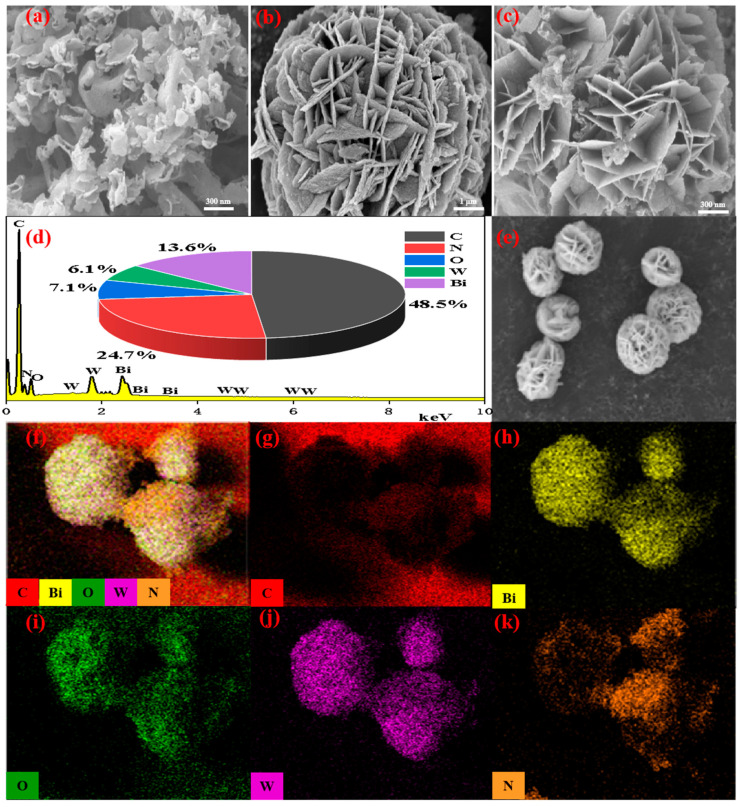
SEM images of (**a**) g-C_3_N_4_, (**b**) Bi_2_WO_6_ and (**c**) BC-3, (**d**) EDS spectrum, (**e**) electronic image, and (**f**–**k**) elemental mapping of BC-3.

The microstructure and element analysis of the obtained catalysts were analyzed by FE-SEM and EDS. Figure 3a manifested that g-C_3_N_4_ had an obvious fold-layered structure. As could be observed, Bi_2_WO_6_ (Figure 3b) showed a compact flower-like structure of a micron size formed by nanosheets. As shown in Figure 3c, g-C_3_N_4_ was uniformly incorporated on the surface and cavity of Bi_2_WO_6_. The EDS and element mapping of the Bi_2_WO_6_/g-C_3_N_4_ heterojunction was displayed in Figure 3d–k. EDS patterns proved the presence of Bi, W, O, N and C in BC-3, and the contents of major elements were 48.5% (C), 24.7% (N), 7.1% (O), 6.1% (W), and 13.6% (Bi) (Figure 3d), respectively. The results were basically consistent with the characterization of XPS. The element maps further demonstrated the successful synthesis of the Bi_2_WO_6_/g-C_3_N_4_ photocatalyst.

### 2.2. Optical and Photoelectrochemical Properties

#### 2.2.1. UV-Vis DRS Analysis

**Figure 4:**
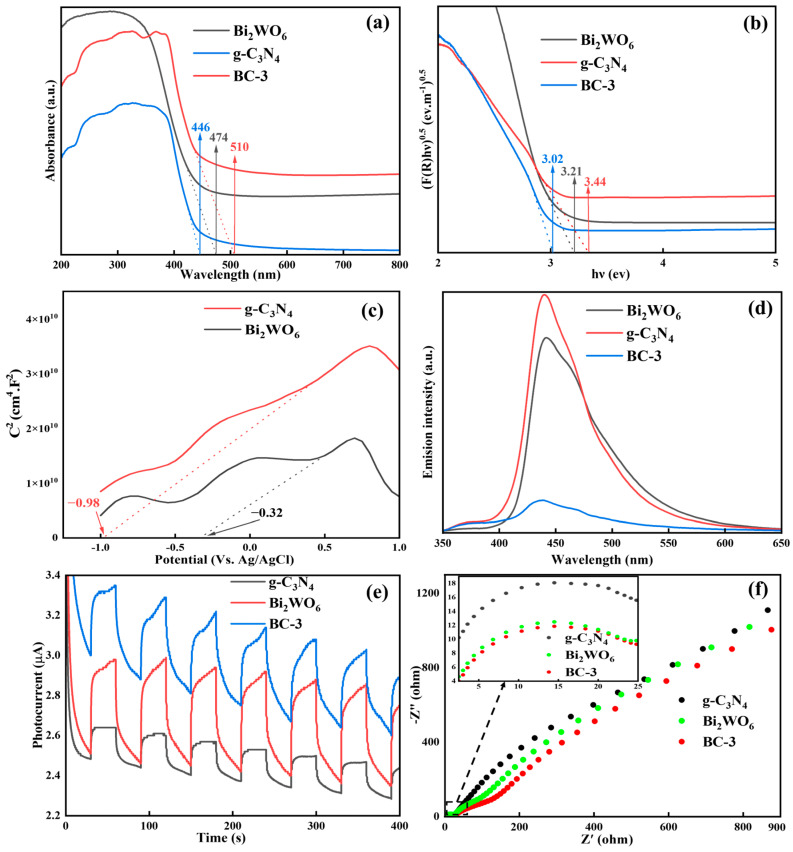
(**a**) UV-vis DRS, (**b**) bandgap energy, (**c**) Mott–Schottky, (**d**) PL spectra, (**e**) i-t curves, and (**f**) EIS.

The light absorption performance of the photocatalyst was investigated using UV-vis DRS spectroscopy. As illustrated in Figure 4a, the absorption edges were about 446 nm (g-C_3_N_4_), 474 nm (Bi_2_WO_6_) and 510 nm (BC-3), respectively. The construction of the heterojunction broadened the absorption edge of the photocatalyst, which might improve its photocatalytic performance. The band gap energies (E_g_) could be estimated using the Kubelka–Munk equation. As exhibited in Figure 4b, the E_g_ of g-C_3_N_4_, Bi_2_WO_6_, and BC-3 were determined as 3.44, 3.21, and 3.02 eV, respectively [29]. The lower the E_g_ value of the catalyst, the higher the catalytic performance. In light of this, BC-3 might possess the excellent photocatalytic performance. Moreover, the flat band potential (E_fb_) was directly determined on the basis of the Mott–Schottky diagram. As exhibited in Figure 4c, the E_fb_ values of g-C_3_N_4_ and Bi_2_WO_6_ were −0.32 and −0.98 eV (vs. Ag/AgCl), which corresponded to −0.22 and −0.88 eV (vs. NHE), respectively. Usually, the conduction band potential (E_CB_) value was 0.1 eV higher than that of E_fb_ in n-type semiconductors [15]. Therefore, the valence band potential (E_VB_) values of g-C_3_N_4_ and Bi_2_WO_6_ were estimated as 3.32 and 2.43 eV, respectively.

#### 2.2.2. PL Analysis

The separation ability of e^−^-h^+^ pairs were evaluated by PL spectra. As illustrated in Figure 4d, the PL intensity of the samples followed this sequence: g-C_3_N_4_ > Bi_2_WO_6_ > BC-3, demonstrating that the heterostructure of Bi_2_WO_6_/g-C_3_N_4_ could availably suppress the recombination of e^−^-h^+^ pairs. Contrasted with g-C_3_N_4_, the luminous intensity of BC-3 was reduced by 95.7%, which was because of the effective transport of the photo-induced electron from g-C_3_N_4_ to Bi_2_WO_6_ in the BC-3 composite. Generally speaking, the weaker the PL strength, the higher the catalytic performance [30].

#### 2.2.3. Electrochemical Analysis

The photocurrent response (i-t) curve was used to further study the photocurrent density by an electrochemical workstation. As exhibited in Figure 4e, the photocurrent density of BC-3 was the best, which demonstrated that the construction of heterojunctions could accelerate the carrier migration and ultimately improve the catalytic activity. The charge separation and transfer capability of the photocatalyst was measured by EIS. As exhibited in Figure 4f, the arc radius of BC-3 was smaller than Bi_2_WO_6_ and g-C_3_N_4_, indicating that BC-3 owned the outstanding charge separation and transfer performance [31]. In addition, the electron lifetime (*τ*) was closely related to the frequency (*f*), and could be estimated based on Equation (1):

(1) $$\tau =\frac{1}{2\pi f}$$

The electron lifetime of BC-3 (0.0504 ms) was longer than Bi_2_WO_6_ (0.0193 ms) and g-C_3_N_4_ (0.0159 ms), demonstrating that BC-3 had the lowest electron–hole pair recombination rate and charge transfer resistance. These results were consistent with the i-t curve.

#### 2.2.4. BET Analysis

**Figure 5:**
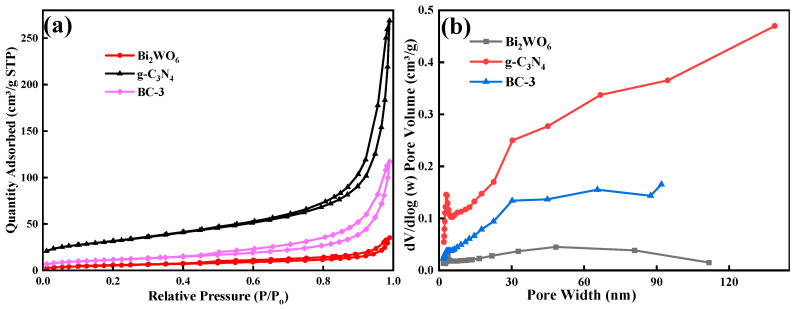
(**a**) N_2_ adsorption–desorption isotherms and (**b**) distribution of pore size plots of g-C_3_N_4_, Bi_2_WO_6_, and BC-3.

The SSA of the prepared samples was estimated by the adsorption–desorption isotherm of N_2_. The catalysts exhibited typical type-III isotherms with distinct H3 hysteresis loops (Figure 5a). The SSA of photocatalysts was 111.67 m^2^/g (g-C_3_N_4_), 80.28 m^2^/g (BC-3), and 20.00 m^2^/g (Bi_2_WO_6_), respectively. The SSA of BC-3 was slightly reduced by the introduction of Bi_2_WO_6_, which might be the effective combination of Bi_2_WO_6_; g-C_3_N_4_ took up its internal space of Bi_2_WO_6_ [16]. Pore size distribution curves (Figure 5b) demonstrated that the pore sizes were 3.54 nm (g-C_3_N_4_), 2.66 nm (Bi_2_WO_6_), and 3.08 nm (BC-3), respectively, which implied that the catalysts were mesoporous materials.

### 2.3. Comparative Tests on TC Removal

**Figure 6:**
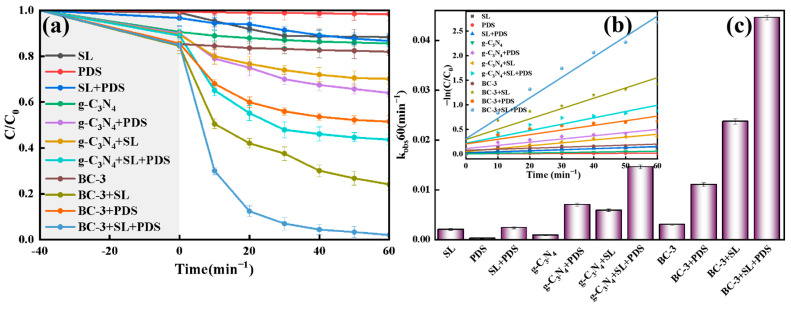
(**a**) TC decontamination degradation curves, (**b**) pseudo first-order kinetic plots and (**c**) K_obs_ in different reaction processes.

The decomposition of TC was discussed in different systems. As displayed in Figure 6a, the decomposition rate of TC was very low in SL (11.6%), illustrating that TC was stable and easy to remain in nature. The removal ratio of TC in SL/PDS, g-C_3_N_4_/SL, and g-C_3_N_4_/SL/PDS systems was 13.3%, 29.9%, and 56.4%, respectively, indicating that g-C_3_N_4_ had a certain activation ability in PDS in the TC degradation. The maximum value achieved 98.0% in the BC-3/SL/PDS system; the results demonstrated that building a heterojunction was beneficial for the removal of TC. As exhibited in Figure 6b, the decomposition of TC deferred to the pseudo first-order kinetic model (R^2^ > 0.532). The apparent reaction rate constants (k_obs_) of TC in different reaction processes were exhibited in Figure 6c; the maximum k_obs_ (0.0446 min^−1^) in the BC-3/SL/PDS system was 3.05 times that of g-C_3_N_4_/SL/PDS (0.0146 min^−1^).

### 2.4. Parameters Impacting on TC Degradation

The effect of the Bi_2_WO_6_ doping ratio, catalyst dosage, initial pH value, TC concentration, PDS dose, and co-existing components on the decontamination rate of TC in the BC-3/SL/PDS system was studied and discussed in detail.

#### 2.4.1. Effect of the Bi_2_WO_6_ Doping Ratio

**Figure 7:**
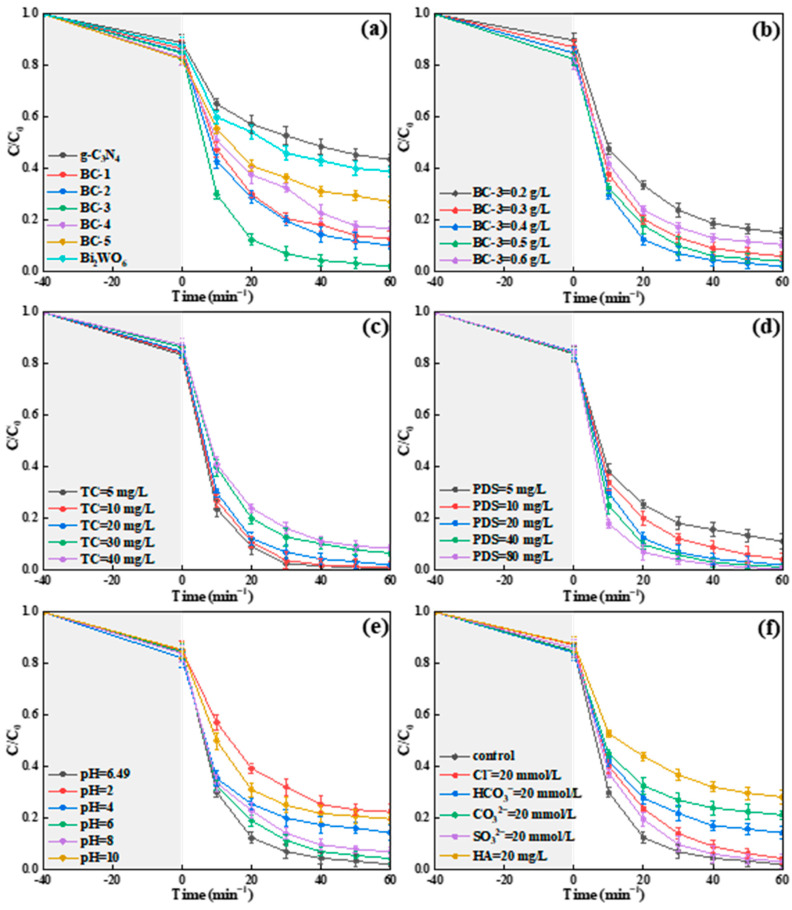
Effects of (**a**) Bi_2_WO_6_ doping ratio, (**b**) BC-3 dosage, (**c**) TC concentration, (**d**) PDS dose, (**e**) initial solution pH, and (**f**) co-existing components in TC degradation.

The experiment was carried out to assess the influence of the Bi_2_WO_6_ doping ratio on the TC decomposition in conjunction with PDS (20 mg/L) under SL. As exhibited in Figure 7a, the decomposition ratios of TC were 56.4% and 61.1% with g-C_3_N_4_ and Bi_2_WO_6_, respectively. Moreover, the removal efficiencies of BC-1, BC-2, BC-3, BC-4, and BC-5 for TC were 87.3%, 89.9%, 98.0%, 83.4%, and 72.8%, respectively. The Bi_2_WO_6_/g-C_3_N_4_ composites had a stronger photocatalysis–persulfate activation for TC degradation than g-C_3_N_4_ and Bi_2_WO_6_. With the increase in the Bi_2_WO_6_ doping ratio, the removal rates of TC first increased and then decreased in Bi_2_WO_6_/g-C_3_N_4_/SL/PDS systems. In particular, the effective transfer of the photoinduced electron in the Bi_2_WO_6_/g-C_3_N_4_ composite was conducive to degrade TC. However, the excessive Bi_2_WO_6_ hindered the transfer of photo-induced carriers while the Bi_2_WO_6_ doping ratio was higher than 4:6. Thus, the optimal Bi_2_WO_6_ doping ratio was 4:6 (BC-3), and the BC-3/SL/PDS system was used in the subsequent degradation experiment.

#### 2.4.2. Influence of the Catalyst BC-3 Dosage

The dosage of BC-3 (0.2–0.6 g/L) was used to explore its influence on the decomposition rate of TC. As exhibited in Figure 7b, the removal ratios of TC were 84.9%, 94.0%, 98.0%, 95.9%, and 89.5%, respectively, when the concentrations of BC-3 were 0.2, 0.3, 0.4, 0.5, and 0.6 g/L. With the increase in BC-3, the degradation ratio increased significantly, while the BC-3 usage ranged from 0.2 to 0.4 g/L. The larger SSA and more redox-active sites caused by the increased catalysts were conducive to activate the PDS. Nevertheless, the decomposition ratio of TC was inhibited when the dose of BC-3 was more than 0.4 g/L, probably because the increase in turbidity intensified the light dispersion [32]. Therefore, the concentration of BC-3 was confirmed as 0.4 g/L.

#### 2.4.3. Influence of TC Concentration

The influence of TC concentration (5–40 mg/L) on the photocatalytic performance was discussed over the BC-3/SL/PDS system. As revealed in Figure 7c, the decomposition efficiency of TC was about 99.5% (5 mg/L), 98.7% (10 mg/L), 98.0% (20 mg/L), 93.3% (30 mg/L), and 91.4% (40 mg/L), respectively. It is worth noting that the decomposition ratio of TC (5 and 10 mg/L) reached about 98% in 40 min. With the content of TC increased from 20 to 40 mg/L, the decomposition rate decreased slightly. A reasonable explanation was that the reactive oxygen species (ROSs) produced in the BC-3/SL/PDS system were not sufficient to decompose the excess TC. Thus, 20 mg/L of TC was used to conduct the follow-up study.

#### 2.4.4. Influence of PDS Dose

The impact of PDS dose on the decomposition efficiency of TC was discussed in the BC-3/SL/PDS system. As demonstrated in Figure 7d, the decomposition ratios of TC were 88.8% (5 mg/L), 95.8% (10 mg/L), 98.0% (20 mg/L), 98.8% (40 mg/L), and 99.2% (80 mg/L), respectively. The decomposition ratio of TC was continuously improved with the increase in PDS dose, which was because there were more active factors generated by PDS. The PDS dose was chosen as 20 mg/L in the follow-up experiment.

#### 2.4.5. Influence of Initial Solution pH

The initial pH was studied in the BC-3/SL/PDS system. TC exhibits TCH^3+^, TCH_2_^0^, and TCH^¯^ or TC^2−^, respectively, when the value of pH was less than 3.3, between 3.3 and 7.7, and more than 7.7 [25]. The zero charge points (pH_PZC_) of BC-3 was 6.09. Therefore, the charge of BC-3 was positive when the pH values were 2, 4, and 6, and the surface charge was negative when the pH values were 8 and 10. As exhibited in Figure 7e, the removal efficiency of TC was 77.6%, 85.7%, 95.7%, 98.0%, 93.2%, and 80.8%, respectively, when the value of pH was 2, 4, 6, 6.49 (unadjusted), 8, and 10. The repulsion force between TC and BC-3 was harmful to the degradation of TC, while the value of pH was less than 6.09 or more than 7.7. However, the vanished repulsive force, 6.09 ≤ pH ≤ 7.7, promoted the adsorption of TC on the interface of BC-3, which was beneficial to the catalytic oxidation of TC. Hence, the value of the initial solution’s pH was determined as 6.49 (unadjusted) in the BC-3/SL/PDS system.

#### 2.4.6. Influence of Co-Existing Components

HA, CO_3_^2−^, HCO_3_^−^, Cl^−^, and SO_4_^2−^ widely existed in water, and had different impacts on the catalytic oxidation of contaminant. Hence, the influence of co-existing components on the decomposition of TC was assessed in the BC-3/SL/PDS system. As illustrated in Figure 7f, the inhibition effects of different co-existing components on the TC degradation were in sequence: HA > CO_3_^2−^ > HCO_3_^−^ > Cl^−^ > SO_4_^2−^. The decomposition ratio of TC was 71.8% (HA), 78.8% (CO_3_^2−^), 85.8% (HCO_3_^−^), 95.7% (Cl^−^), and 97% (SO_4_^2−^), respectively. HA exhibited the most obvious inhibitory action for TC degradation, which was because of the suppressed light absorption in the BC-3/SL/PDS system. Moreover, the competitive adsorption of CO_3_^2−^ and S_2_O_8_^2−^ to binding sites on the surface of BC-3 limited the decomposition of TC. SO_4_•^−^ and the hydroxyl radical (•OH) captured by HCO_3_^−^ were harmful to the oxygenolysis of TC [33]. Interestingly, Cl^−^ and SO_4_^2−^ exhibited an insignificant influence on the TC degradation. The result confirmed that the BC-3/SL/PDS system presented great merits for the degradation of TC in a complex water environment.

#### 2.4.7. Stability Assessment

**Figure 8:**
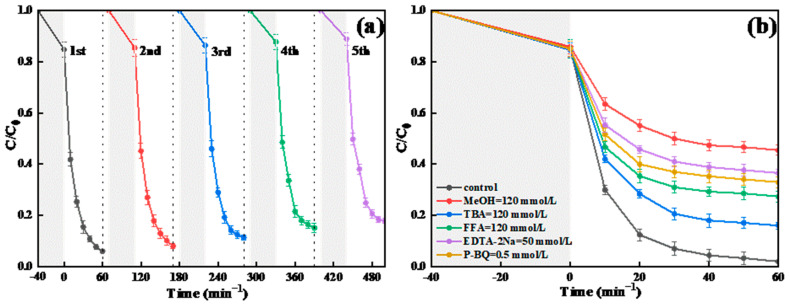
(**a**) Cyclic test of BC-3 and (**b**) radical trapping experiment of BC-3/SL/PDS system.

The stability of BC-3 was tested by means of a multiple-cycle test. As displayed in Figure 8a, the decomposition ratio of TC remained at 82.2% after 5 cycles under the best experimental conditions, which might be because the reactive sites of the catalyst surfaces were occupied by the byproducts. The cycling test showed that BC-3 possessed a high physicochemical stability for the TC degradation in the BC-3/SL/PDS system. Further, the catalytic performance of Bi_2_WO_6_/g-C_3_N_4_ was also compared in this research and recent reports, as illustrated in Table 1. The BC-3/SL/PDS system represented a better catalytic performance of TC than other processes. Therefore, the BC-3/SL/PDS system was a satisfactory process to use when treating antibiotic wastewater.

#### 2.4.8. ROSs and Reaction Mechanisms in BC-3/SL/PDS System

Free radical quenching experiments were applied to discern ROSs of TC degradation in the BC-3/SL/PDS system. p-benzoquinone (P-BQ), disodium EDTA (EDTA-2Na), furfuralcohol (FFA), methyl alcohol (MeOH), and tert butyl alcohol (TBA) were employed to quench O_2_•^−^, h^+^, ^1^O_2_, SO_4_•^−^, and •OH, •OH, respectively. As displayed in Figure 8b, the decomposition ratio of TC was 65.0% (P-BQ), 61.4% (EDTA-2Na), 70.5% (FFA), 54.5% (MeOH), and 84.0% (TBA), respectively. EDTA-2Na showed the apparent inhibition on TC degradation, which indicated that h^+^ made great contributions to the degradation of TC. Similarly, the oxidation reactions were greatly suppressed after adding P-BQ and FFA, which certified that O_2_•^−^ and ^1^O_2_ were the primary ROSs for the decomposition. Moreover, the degradation efficiency declined substantially to 54.5% after adding MeOH, which verified the existence of both SO_4_^•−^ and •OH species. However, the decomposition ratio of TC decreased slightly (84.0%) when TBA was added, which demonstrated that •OH was not the dominant ROSs. In conclusion, h^+^, O_2_•^−^, SO_4_•^−^, and ^1^O_2_ were the main ROSs in the BC-3/SL/PDS system, while the TC degradation was relatively little influenced by •OH.

**Figure 9:**
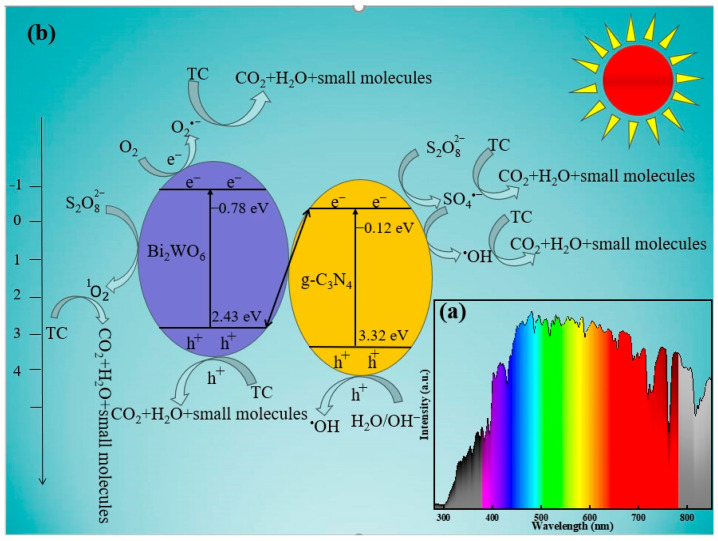
(**a**) Spectrogram of solar light, (**b**) schematic diagram of photo-generating carrier transfer, and free radical generation in BC-3/SL/PDS system under solar light irradiation.

The possible reaction mechanism for TC degradation by the photocatalysis–persulfate activation was proposed in the BC-3/SL/PDS system (Figure 9b). The E_VB_ of g-C_3_N_4_ and Bi_2_WO_6_ were 3.32 eV and 2.43 eV, and the E_CB_ were −0.12 eV and −0.78 eV (vs. NHE) (Figure 5). Thus, g-C_3_N_4_ and Bi_2_WO_6_ were motivated to generate e^−^-h^+^ pairs by the solar light irradiation whose spectrum was exhibited in Figure 9a. The Bi_2_WO_6_/g-C_3_N_4_ heterojunction was divided into a type-Ⅱ or Z-scheme heterojunction based on the transferred behavior of e^−^ between g-C_3_N_4_ and Bi_2_WO_6_. Since the E_CB_ (−0.78 eV) of Bi_2_WO_6_ was more negative than O_2_/O_2_•^−^ (−0.33 eV vs. NHE) [16], e^−^ in the conduction band of Bi_2_WO_6_ could react with O_2_ to produce O_2_•^−^. In addition, the E_VB_ (+3.32 eV) of g-C_3_N_4_ was higher than •OH/H_2_O (+2.40 eV vs. NHE) [15], and h^+^ in the valence band of g-C_3_N_4_ was able to react with H_2_O/OH^−^ to generate •OH. These results agreed with free radical quenching experiments, confirmed that BC-3 belonged to the Z-scheme heterojunctions. Moreover, PDS was able to break up into SO_4_•^−^ (Equation (4)), and reacted with OH^−^ to produce ^1^O_2_ for the activation of BC-3 according to Equation (6). And, ^•^OH was generated by SO_4_•^−^ and OH^−^ based on Equation (5) [6].

In the BC-3/SL/PDS system, O_2_•^−^, SO_4_•^−^ and •OH free radicals; h^+^; and ^1^O_2_ non-free radicals could decompose TC into CO_2_, H_2_O, and small molecules. The possible reactions were described in Equations (2)–(8).

BC-3 + solar light → BC-3 (h^+^ + e^−^) (2)

O_2_ + BC-3 (e^−^) → O_2_•^−^ (3)

S_2_O_8_^2−^ + BC-3 → SO_4_•^−^ + SO_4_^2−^ (4)

SO_4_•^−^ + BC-3 + OH^−^ → •OH + SO_4_^2−^ (5)

S_2_O_8_^2−^ + BC-3 + 2OH^−^ → ^1^O_2_ + 2SO_4_^2−^ + H_2_O (6)

H_2_O/OH^−^ + BC-3 (e^−^) → •OH (7)

TC + h^+^/O_2_•^−^/SO_4_•^−^/•OH/^1^O_2_ → H_2_O + CO_2_ + small molecules (8)

## 3. Experimental Section

### 3.1. Preparation of Bi_2_WO_6_/g-C_3_N_4_

g-C_3_N_4_ was obtained using urea using the thermal polymerization method, as described in our previous study [38]. Bi_2_WO_6_/g-C_3_N_4_ was prepared via a hydrothermal process. Simply stated, 0.98 g Bi(NO_3_)_3_·5H_2_O was put into 10 mL CH_3_COOH under magnetic stirring for 20 min (labeled as A). Then, 0.33 g Na_2_WO_4_·2H_2_O and a number of quality of g-C_3_N_4_ were successively poured into 50 mL of deionized water by an ultrasonic wave for 20 min (labeled as B). Next, solution B was blended with A and dispersed uniformly by an ultrasonic wave for 20 min. The obtained mixture was subsequently put in a high-pressure reactor and remained at 180 °C for 3 h. The resulting product was centrifuged and then washed with deionized water. Finally, the solid powder was naturally dried and stored for later use. Bi_2_WO_6_/g-C_3_N_4_ with various ratios of g-C_3_N_4_ and Bi_2_WO_6_ at 4:6, 5:5, 6:4, 7:3, and 8:2 was expressed as BC-1, BC-2, BC-3, BC-4, and BC-5, respectively. Pure Bi_2_WO_6_ was also prepared using the same procedure with the absence of g-C_3_N_4_.

### 3.2. Characterization

The molecular information of the catalyst was tested by Fourier transform infrared spectroscopy (FT-IR, Cary630 (Agilent, Palo Alto, CA, USA)). The crystalline form of the photocatalyst was characterized by an X-ray diffractometer (XRD, Rigaku/Smart Lab SE (Rigaku, Tokyo, Japan)). The element and structure of the photocatalyst were determined by X-ray photoelectron spectroscopy (XPS, Thermo Scientific ESCALAB Xi+ (Thermo Fisher Scientific, Waltham, MA, USA)). The morphology and element composition of the photocatalyst were studied by a field emission scanning electron microscopy (FE-SEM, Sigma300 (Carl Zeiss, Oberkochen, Germany)) equipped with an energy dispersive X-ray spectroscopy (EDS, Sigma300 (Carl Zeiss, Oberkochen, Germany)). The light absorption characteristic was tested by ultraviolet-visible diffuse reflection spectroscopy (UV-vis DRS, Cary7000 (Agilent, Palo Alto, USA)). The photoelectric properties were studied with photo-luminescence (PL, F4600 (Hitachi, Tokyo, Japan)) and electrochemical systems (CHI E660 (CH Instruments, Inc., Tennison Hill Drive Austin, TX, USA)). Electrochemical tests included electrochemical impedance spectra (EIS), Mott–Schottky and photocurrent spectroscopy, respectively. The specific surface area (SSA) was identified by a Brunauer–Emmett–Teller (BET, Belsorp Maxll (Micromeritics, Atlanta, GA, USA)).

### 3.3. Photocatalytic Degradation

The performance of Bi_2_WO_6_/g-C_3_N_4_ composites was studied via the decomposition experiment of TC under solar light (103 ± 3 mW/cm^2^, 26 ± 1 °C) for 60 min. Briefly, 20 mg of samples was added into 20 mg/L TC (50 mL). The solution-containing catalyst was stirred (300 r/min) in the dark for 40 min. The beaker was then illuminated by solar light after adding PDS. At a certain time, 1.5 mL solution filtered with a microfiltration membrane (0.45 μm) was used to estimate the concentration of TC based on the absorbance at the maximum wavelength (358 nm) by a UV-vis spectrophotometer (UV1901PC).

## 4. Conclusions

In summary, the Z-scheme heterojunction Bi_2_WO_6_/g-C_3_N_4_ was successfully obtained by a single-step hydrothermal method for 3 h, and used as an activator of PDS in conjunction with SL to decompose TC. The removal ratio of TC was 98.0% at the optimum experimental conditions (pH = 6.49, BC-3 = 0.4 g/L, TC = 20 mg/L and PDS = 20 mg/L), and the most suitable ratio of g-C_3_N_4_ to Bi_2_WO_6_ was 6:4. The k_obs_ was 0.0446 min^−1^ in the BC-3/SL/PDS system, which was 3.05 times than that of g-C_3_N_4_/SL/PDS (0.0146 min^−1^). BC-3 exhibited an excellent performance for the efficient electron transfer in the photocatalytic oxidative experiment of TC removal. h^+^, O_2_•^−^, SO_4_•^−^, and ^1^O_2_ generated by the synergy of BC-3, SL, and PDS promoted the degradation of TC. HA represented the strongest suppression effect (decreased by 26.2%) on the TC degradation in the test of co-existing components, indicating that the BC-3/SL/PDS system possessed a strong anti-interference capacity. Therefore, this study not only provided a novel synthesis method of the Bi_2_WO_6_/g-C_3_N_4_ Z-scheme heterojunction, but also provided a practical technique for treating antibiotic wastewater.

## Figures and Tables

**Table 1:** The comparison between different Bi_2_WO_6_/g-C_3_N_4_ catalysts into TC removal.

Process	Antibiotic	Operating Conditions	Degradation Rate	k_obs_ (min^−1^)	Ref.
CNQDs/BWO + vis	TC	[pH]: - [Catalyst]: 1.0 g/L [Nor]: 20 mg/L [Time]: 60 min	87.0%	-	[34]
flower-like dual Z-scheme BiSI/Bi_2_WO_6_/g-C_3_N_4_ + vis	TC	[pH]: - [Catalyst]: 0.6 g/L [Nor]: 20 mg/L [Time]: 60 min	90.0%	-	[35]
Agx/Bi_2_WO_6_/g-C_3_N_4_ + vis	TC	[pH]: - [Catalyst]: 0.5 g/L [Nor]: 15 mg/L [Time]: 60 min	81.4%	0.028 min^−1^	[36]
Bi_2_WO_6_/BiOI/g-C_3_N_4_ nanoparticles + vis	TC	[pH]: 9.0 [Catalyst]: 1.0 g/L [Nor]: 20 mg/L [Time]: 120 min	94.5%	-	[37]
Bi_2_WO_6_/g-C_3_N_4_ + SL + PDS	TC	[pH]: 6.49 [Catalyst]: 0.4 g/L [Nor]: 20 mg/L [Time]: 60 min	98.0%	0.0446 min^−1^	This work

## Data Availability

Data are contained within the article.
